# Investigation of Microstructure and Wear Properties of Precipitates-Strengthened Cu-Ni-Si-Fe Alloy

**DOI:** 10.3390/ma16031193

**Published:** 2023-01-30

**Authors:** Chun-Hao Peng, Po-Yu Hou, Woei-Shyang Lin, Pai-Keng Shen, Hao-Hsuan Huang, Jien-Wei Yeh, Hung-Wei Yen, Cheng-Yao Huang, Che-Wei Tsai

**Affiliations:** 1 Department of Materials Science and Engineering, National Tsing Hua University, Hsinchu 30013, Taiwan; 2 High Entropy Materials Center, National Tsing Hua University, Hsinchu 30013, Taiwan; 3 Department of Materials Science and Engineering, National Taiwan University, Taipei 10617, Taiwan

**Keywords:** sliding wear, intermetallic, high Ni/Si ratio, precipitation hardening

## Abstract

Based on multi-component alloys using precipitation hardening, a Cu-Ni-Si-Fe copper alloy was prepared and studied for hardness, electrical conductivity, and wear resistance. Copper Nickel Silicon (Cu-Ni-Si) intermetallic compounds were observed as precipitates, leading to an increase in mechanical and physical properties. Further, the addition of Fe was discussed in intermetallic compound formation. Moreover, microstructures, age hardening, and dry sliding wear resistances of the present alloy were analyzed and compared with C17200 beryllium copper. The results showed that the present alloy performed extraordinarily, with 314 HV in hardness and 22.2 %IACS in conductivity, which is almost similar to C17200 alloy. Furthermore, the dry sliding wear resistance of the present alloy was 2199.3 (m/MPa·mm^3^) at an ambient temperature, leading to an improvement of 208% compared with the C17200 alloy.

## 1. Introduction

Copper and copper alloys are widely used in industries owing to their desirable properties such as excellent electrical and thermal conductivity, as well as good mechanical properties [1]. Among copper alloys, precipitation hardened copper alloys play an important role in industrial applications owing to their improved mechanical and physical properties [2,3,4].

According to the results from the solubility of solutes under different temperatures, a super-saturated solid solution forms at an ambient temperature. Following the aging process, precipitation hardening occurs, thereby improving the hardness and electrical conductivity. According to the Orowan strengthening theory, the amount of strengthening varies with the fraction of precipitates and their size [5,6,7,8,9]. Based on the scattering mechanisms [10,11,12,13], due to the second phase precipitation, the solute atoms dissolved in the copper-rich matrix are reduced, thereby decreasing the degree of electron scattering and increasing the conductivity of the alloy [14,15]. Considering C17200 beryllium copper as an example, a super-saturated solid solution can be formed by quenching from 886 °C to 25 °C. Precipitation hardening occurs owing to the subsequent aging between 400 and 500 °C, and the hardness and electrical conductivity of the C17200 alloy increase to approximately 400 HV and 20~25 %IACS [16,17], resulting in it being widely used in industrial applications such as electrical connectors, switches, etc. [18].

Although the properties of beryllium copper are superior, its exorbitant cost and toxicity during processing lead to cost and safety concerns [19]. Hence, Be-free copper alloys are required [20]. The precipitation-strengthened copper alloys that are improved by adding different elements include Cu-Fe-P [21,22,23], Cu-Ni-Si [24,25], Cu-Zn-Sn [26,27], and Cu-Cr-Zr [28,29,30]. Several researchers have found that Cu-Ni-Si system alloys have outstanding properties and huge development potential. Cu-Ni-Si system alloys have an excellent electrical and thermal conductivity, as well as good mechanical strength [31,32]. Researchers have found that the high strength and electrical properties of Cu-Ni-Si system alloys contribute to the uniform distribution of nanoscale δ-Ni_2_Si to the matrix [33,34,35,36,37,38]. Furthermore, when the weight ratio of Ni/Si is between four and five, the precipitation has the best balance of strength and electrical conductivity [39]. After cold work and aging heat treatment, the precipitation of δ-Ni_2_Si strengthens rapidly, enabling the alloys to reach peak hardness in 1 h [24,39]. Simultaneously, the large amount of (Ni + Si) added causes the formation of micron-scale intermetallic compounds after casting [40]. In addition, other elements such as chromium, titanium, silver, or magnesium are added to promote the mechanical properties of the Cu-Ni-Si alloy. These intermetallic compounds of Cu-Ni-Si with a higher Ni-Si ratio or with the addition of other elements cannot be re-dissolved into the matrix. However, the influence of these intermetallic compounds on the mechanical properties is still not quite clear. Furthermore, only few studies demonstrate the role of iron addition in grain refinement [41,42]. Extensive research has not been carried out on the function of iron addition on precipitation.

In this study, a Cu-Ni-Si-Fe copper alloy without beryllium element was studied, with a Ni/Si weight ratio of 4.1 according to recent research results. To understand how intermetallic compounds and iron affect the wear property in ambient temperatures, Cu_86.5_Ni_7.4_Si_3.8_Fe_1.1_ alloys with high nickel and silicon contents were prepared and an investigation on microstructures’ relations to wear properties was carried out.

## 2. Materials and Methods

The nominal composition of C17200 is approximately between 1.8~2.0 wt.% Be and balanced with Cu. In comparison, the nominal composition of present Cu-Ni-Si-Fe alloy is Cu_86.5_Ni_7.4_Si_3.8_Fe_1.1_ (Cu86.5) in molar ratio. Pure copper, nickel, silicon, and iron were prepared and melted using vacuum arc remelting (VAR) under a protective argon atmosphere. After casting, the as-cast ingot was homogenized at 900 °C for 6 h by water-quenching and subsequently cold rolled by 40%. The as-rolled strip was aged between 450 and 500 °C for 0.5, 1, and 3 h, respectively, and subsequently water-quenched.

The microstructures of the present alloy were observed using JEOL JSM-IT100 scanning electron microscope (SEM), energy dispersive spectrometer (EDS), and FEI Tecnai G2 F20 transmission electron microscope (TEM). The phases of the alloy were identified using Bruker D2 Phaser X-ray diffractometer (XRD) with Cu-Kα radiation (λ = 1.5405 Å) and a scanning velocity of 0.03° per second. The Rietveld refinement analysis method was used in calculating δ-Ni_2_Si lattice constant from XRD diffraction peak, while the calculation and fitting were performed using the Maud software. Hardness test was carried out on Mitutoyo HV 100 Vickers hardness tester using a 5 kg load and holding time for 12 s, while considering the average of 6 values.

Electrical conductivity was measured at ambient temperature using Fischer Sigmascope SMP350 conductivity meter, considering the average of 6 values. Wearing tests were performed on a pin-on-disk device with a load of 0.58 MPa, a wearing velocity of 0.5 m per second, and a duration time of 3 h. The counterparts of the wearing tests were SKD 11 tool steels (~700 HV). The density and volume loss of the sample were calculated and measured using Archimedes’ principle. The area fraction of inclusions was analyzed using ImageJ software ver.1.53.

## 3. Results and Discussion

### 3.1. Mechanical and Electricaal Properties

**Figure 1:**
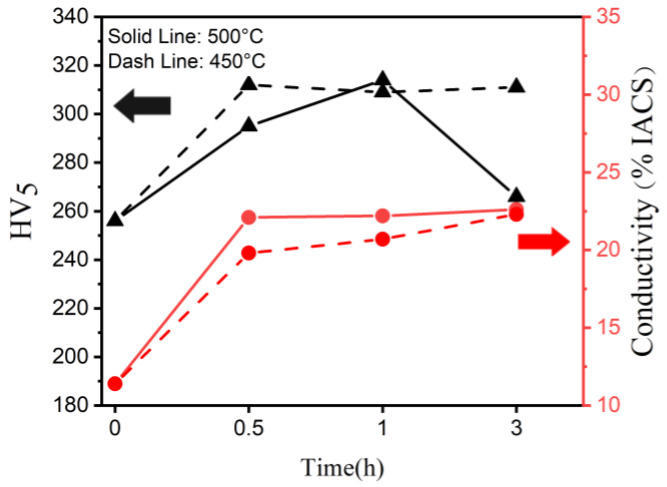
Aging curve of Cu_86.5_ at 450 and 500 °C from a 40% cold rolling for 0.5, 1, and 3 h.

After arc melting, homogenization at 900 °C for 6 h, cold rolling at 40%, and aging heat treatment between 450 and 500 °C for different durations were performed. Figure 1 shows the hardness and conductivity changes with time after cold rolling and aging at 450 and 500 °C. Under both 450 and 500 °C, the peak hardness of 312 HV and 314 HV was attained in 0.5 and 1 h, respectively. However, if the aging continued under 500 °C, owing to over-aging, the hardness considerably decreased after 3 h, remaining 266 HV. Compared with 500 °C, aging at 450 °C reached peak hardness in 30 min rapidly, while maintaining nearly the same hardness for 3 h. The aging curve was quite similar to other works [43]. Under 450 and 500 °C, the conductivity increased with the aging time, reaching 22.2 %IACS and 20.7 %IACS in 1 h, respectively.

This is related to the previously mentioned electron scattering of the elements redissolved in the Cu matrix [11,12,14,44]. During the aging heat treatment, the nanoscale precipitates (discussed later) were released, thereby purifying the matrix. As a result, the conductivity increased steadily under both 450 and 500 °C. Overall, the aging precipitation at 500 °C was faster than that at 450 °C and the instances of over-aging were more considerable. This led to the aging softening over 1 h. The hardness and microstructure of the longer aging treatment of the Cu86.5 alloy are listed in the Supplementary file. However, the conductivity increased relatively faster owing to the rapid precipitation at 500 °C. Owing to the subsequent pin-on-disk wearing test, the highest hardness was selected as the test sample; hence, the optimal duration time and temperature of the peak aging heat treatment needed to be found. The wearing test considered the best peak aging state at 500 °C for 1 h, 314 HV, and 22.2 %IACS, as the sample.

### 3.2. Microstructures of Cu_86.5_Ni_7.4_Si_3.8_Fe_1.1_

**Figure 2:**
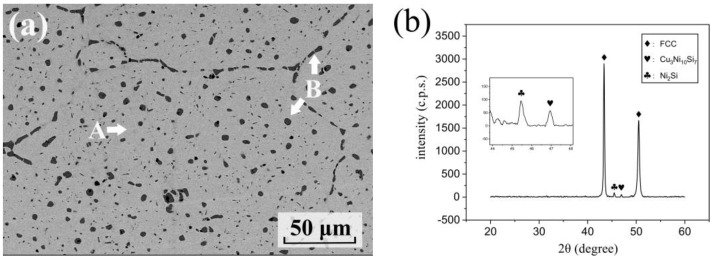
Microstructures of Cu_86.5_ alloy aged at 500 °C for 1 h: (**a**) SEM image (A: Curich matrix and B with arrow: intermetallics) and (**b**) X-ray diffraction pattern.

The microstructures of the as-aged Cu86.5 alloy are shown in Figure 2. Two phases, A and B, are marked in Figure 2a. In Table 1, phase A, is a Cu-rich matrix with 5.3 at.% Ni, 3.8 at.% Si, and 1.1 at.% Fe. Phase B is a Cu-Ni-Si-rich intermetallic phase with 47.7 at.% Ni, 27.0 at.% Si, and 7.6 at.% Fe. In addition, the Fe content in the B phase was relatively high. The as-aged alloys analyzed the crystal structure of phases A and B. From the results of the XRD curves, three peaks can be seen in Figure 2b. The peak at 2θ = 42° indicates an FCC crystal structure, that is, the Cu-rich matrix in Figure 2a. The peak at 2θ = 47° was characterized as B phase intermetallic compound ((Cu, Ni, Si)-rich phase). The intermetallic compound was also found in other studies contributing to the maximum solubility of Si. High content Ni and Si were added, although Ni decreased the solubility of Si in Cu matrix. Consequently, the intermetallic precipitated out in an as-cast state with the addition of more than 5.88% [45,46,47,48]. As seen in Figure 2a, 8 vol.% of intermetallic compounds could be observed in the Cu matrix by ImageJ calculation. This is similar to the results from other research, especially as there was no volume fraction change after further aging. Furthermore, from a thermodynamics viewpoint, the mixing enthalpy of Cu-Si, Ni-Si and Fe-Si were −19, −40, and −35 (kJ/mol), respectively, revealing silicide formation tendencies. The addition of excess Ni reduced the solubility of Si. However, as insoluble Si has a high mixing enthalpy with Cu, Ni, and Fe, it was easy to form intermetallic compounds. In addition, Fe has the highest mixing enthalpy with Si of −35 kJ/mol, which further improves the formation of silicide. Therefore, a relatively high Fe content was present in the B phase. In addition, Hui Xie et al. equally observed the apparition of peak C at 2θ = 45.5°, which was characterized as Ni_2_Si precipitates [49]. After Rietveld refinement analysis, the lattice constant a, b, and c were 7.036 Å, 5.150 Å, and 3.821 Å, respectively. However, Ni_2_Si was not observed during SEM observations as the sizes of these precipitates were in the nanometer range.

### 3.3. Nano Precipitates of Ni_2_Si

**Figure 3:**
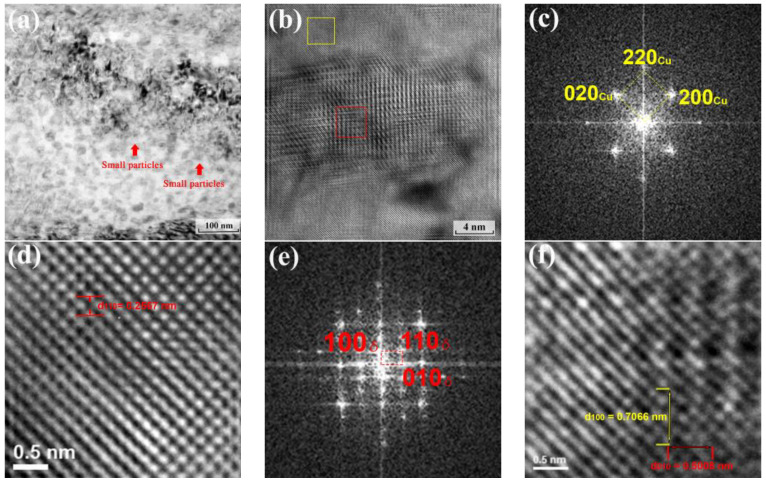
(**a**) TEM images of Cu_86.5_ alloy aged at 500 °C for 1 h bright field image through [100]_Cu_, (**b**) HRTEM image through [100]_Cu_, (**c**) SAED pattern of FCC Cu-rich matrix, (**d**) Lattice image of FCC Cu-rich matrix through [100]_Cu_, (**e**) SAED pattern of δ-Ni_2_Si, and (**f**) Lattice image of δ-Ni_2_Si through [100]_Cu_.

Nano-scaled Ni_2_Si precipitate is the primary precipitation strengthening of Cu-Ni-Si [34,38,39,50]. In this study, the contribution of the B phase to hardness can be disregarded because the volume fraction and microstructure of the B phase remained unchanged during aging. Although the Cu86.5 alloys had high Ni and Si content, the strengthening phase in the Cu-rich matrix phase after aging remained the most dominant, and rapid precipitation strengthening occurred. The nanoscaled Ni_2_Si precipitates were analyzed as seen in Figure 3 using TEM. The bright field image of the as-aged states with 500 °C for 1 h and several small particles indicated as arrows can be observed in Figure 3a. To further confirm the structure and orientation relationship, the high-resolution lattice images in Figure 3b show that, from [100]_Cu_, the size was approximately between 10 and 20 nm in diameter (disc-like shape) and coherent with the Cu-rich matrix. Figure 3c shows the selected area electron diffraction (SAED) pattern of the Cu-rich matrix in the FCC structure (the yellow area in Figure 3b). From the lattice image in Figure 3d, the d-spacing of (110) was 0.2567 nm. Figure 3e shows the SAED pattern of these oval contrasts, that is, the red area in Figure 3b with an electron beam parallel to [100]_Cu_. The diffraction spots along the red square revealed the diffraction pattern of the nanoscaled precipitates, which were δ-Ni_2_Si orthorhombic structures with a = 7.066 Å, b = 5.008 Å, and c = 3.732 Å. The lattice parameters were also marked in the lattice image (Figure 3f), whereby the d-spacing of (100) and (010) corresponded to a and b. This structure and lattice parameters were similar to other works [24,39], and the result corresponded to that of the XRD analysis. The error of the lattice parameter between XRD and TEM were 0.4%, 2.8%, and 2.4%.

### 3.4. Wear Property of C17200 and the as-Aged Cu_86.5_ Alloy

**Figure 4:**
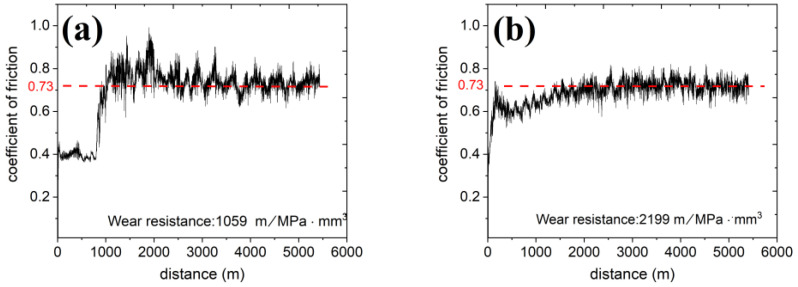
Coefficient of friction of the (**a**) C17200 and (**b**) Cu_86.5_Ni_7.4_Si_3.8_Fe_1.1_ alloy.

The wear curves of C17200 and Cu_86.5_ are shown in Figure 4. A two-stage wear process in the wear test of C17200 can be observed. Initially, a wearing test running-in stage for 1000 m shows a low friction of coefficient of approximately 0.4. After 1000 m of wearing, the friction of coefficient increased sharply and was serrated between 0.68 and 0.83. These had a coefficient of friction of 0.73, even though the wear resistance was quite different. The wear resistance of C17200 and Cu_86.5_ were 1059 m/MPa·mm^3^ and 2199 m/MPa·mm^3^, respectively. The present alloy was twice higher than that of the C17200 alloy. The higher the value, the better the resistance to wear.

The low friction of coefficient can be attributed to the high-hardness surface of the C17200 alloy. Spalling and sintering occurred locally at the C17200 alloy surface, making the normal force concentrate at the less contacted area, the surface becoming much rougher, and the wear resistance worsening. Furthermore, the frequent peeling and formation of the glaze layer led to a drastic rise and fall of the friction coefficient, resulting in a larger amplitude at the curve. Contrarily, the two-stage present alloy was less pronounced, with 0.7 at the beginning, then decreasing to approximately 0.6, increasing to the average, and remaining stable. The decreasing part shows that the contact surface produced something that had a lubricating effect, as will be discussed in the next part.

The wear resistance of both alloys did not follow Archard’s rule, revealing harder materials with less volume loss. The hardness of C17200 and Cu_86.5_ was 400 HV and 314 HV, respectively, under similar coefficient of friction, wear distance, and normal force [51,52].

**Figure 5:**
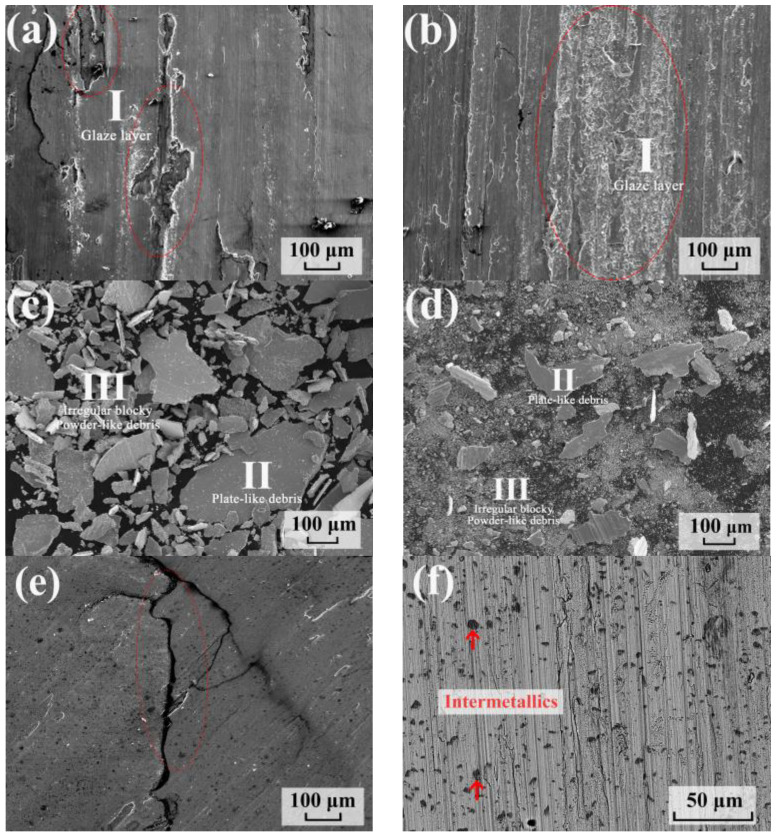
Wear surface and debris of (**a**,**c**) C17200 alloy and (**b**,**d**) the Cu_86.5_ alloy after pin-on-disc test, (**e**) crack on C17200 surface, and (**f**) intermetallic compound left on the surface.

After the wearing test, the microstructure of C17200 and the as-aged Cu_86.5_ alloys are shown in Figure 5. The wearing surface of C17200 (Figure 5a) was mostly flat due to a high hardness. However, some spalling and a few glaze layers (A) could still be seen on the surface. The wearing surface of the Cu_86.5_ alloy after the wearing test resulted in the formation of large areas of oxidation layers on the wearing surface as can be seen in Figure 5b. The composition of the glaze layer (A) is listed in Table 2. The composition of the C17200 glaze layer and the as-aged Cu86.5 alloys were similar to 32.5 at% O and 31 at% O, respectively.

Accumulated debris on the surface and spall from the alloy led to the formation of an oxidation layer (glaze layer) as a result of oxidation and compaction. This generally prevents the surfaces in question from wearing damage. These glaze layers generally peel off after obtaining critical thickness, causing further surface wear [53]. Two types of C17200 alloy debris can be seen in Figure 5c and their compositions are listed in Table 2. These two types include the plate-like debris (B) with 5.2 at.% oxygen and irregular blocky debris (C) with 23.1 at.% oxygen. The composition of B was almost similar to the C17200 alloys, which were spalled directly from the surface during wearing. The composition of C on its part was close to the glaze layer owing to oxidation and sintering. Two types of as-aged Cu_86.5_ alloys debris can be seen in Figure 5d. The larger one is the plate-like debris and the smaller one the powder-like debris. The compositions of these two kinds of debris are also shown in Table 2. The composition of B was close to present alloys and was spalled directly from the surface. However, Ni and Si were not detected in B, implying the intermetallic compounds were wear-resistant, and that they prevented the alloy from wearing. The composition of the powder-like debris (C) was also close to the glaze layer owing to oxidation and sintering during wearing. Furthermore, the smaller debris are advantageous not only for efficiently forming protective glaze layers during wearing, but also for lubricating the interface efficiently. The debris from the glaze layer of C17200 revealed irregular morphology compared with the powder-like debris of the present alloy, resulting in less C17200 oxide layers. Under continuous wearing, the spalling from the brittle surface and the unstable C17200 glaze layer accounted for less glaze layer formation and more volume loss because the glaze layer kept forming and peeling.

In addition to the lesser glaze layer that was formed on the C17200 wear surface, some cracks were also noticed on the surface as seen in Figure 5e. As C17200 had high strengths, it was relatively brittle [54]. In this study, we considered that the brittle surface affected the adhesion strength between the glaze layer and metal interface, making the spalling occur critically. In addition, in Figure 5f, the intermetallic compounds appeared on the surface of the as-aged Cu_86.5_ alloys, and most of the scratches could not pass through the intermetallic compounds, implying that, during the wearing test, the intermetallic compound became more wear-resistant than the matrix and improved the wear-performance of the Cu_86.5_ alloy. Consequently, by adding Fe to Cu-Ni-Si alloys with a higher content of Ni and Si, silicide with insoluble Si could be formed easily, contributing to the formation of wear-resistant intermetallic compounds.

Thus, the low wear resistance of C17200 mainly resulted from the debris and brittle surface. On the one hand, the plate-like debris from the C17200 matrix was larger than that of the Cu_86.5_ alloy, while the surface was brittle. On the other hand, the wear resistance of the as-aged Cu_86.5_ alloys was better than C17200, resulting from the uniform glaze layer and the intermetallic compounds. They served as lubricating and wear-resistant agents effectively during the wear test.

## 4. Conclusions

In this study, high Ni and Si content, with the addition of Fe to the Cu-Ni-Si alloy, was examined. The (Cu, Ni, Si)–rich intermetallic compounds with 8 vol.%, contributing to the mixing enthalpy of Si and nano-scaled δ-Ni_2_Si precipitates, with orthorhombic structures, were identified in a Cu-Ni-Si-Fe alloy aged at 500 °C for 1 h using TEM. The diameter was approximately 10~20 nm and a = 7.066 Å, b = 5.008 Å, and c = 3.732 Å. By maintaining the weight ratio of Ni/Si at 4.1, the hardness and electrical conductivity were increased to 314 HV and 22.2 %IACS by aging hardening, which were 79% and 99% of the C17200 alloys, respectively. Further, wear-resistant intermetallic compounds in the matrix were formed due to the addition of Fe, as well as a high content of Ni and Si. The addition of Fe to the Cu-Ni-Si alloy enhances the formation of intermetallic compound and the wear resistance of Cu_86.5_ alloy.

C17200 and Cu_86.5_ had a similar coefficient of frictions of 0.73, although the wear resistances were 1059 m/MPa·mm^3^ and 2199 m/MPa·mm^3^, respectively. On the one hand, this led to a small oxidation layer and a brittle surface, resulting in worse C17200 wear resistance. On the other hand, a larger oxidation layer area and the intermetallic compounds, which provided excellent protection, increased the wear resistance of the Cu-Ni-Si-Fe alloy to twice higher than that of the C17200 alloy. 

## Figures and Tables

**Table 1:** Composition of Cu_86.5_ alloy aged at 500 °C for 1 h (.at%).

	Cu	Ni	Si	Fe
Overall	Balanced	7.4	3.8	1.1
Cu-rich matrix (A)	Balanced	5.3	2.3	0.7
Intermetallics (B)	Balanced	47.7	27.0	7.6

**Table 2:** Composition of wear surface and debris of C17200 and Cu_86.5_Ni_7.4_Si_3.8_Fe_1.1_.

Alloys	C17200		Cu_86.5_Ni_7.4_Si_3.8_Fe_1.1_				
Elements	Cu	O	Cu	Ni	Si	Fe	O
Glaze layer (I)	67.5	32.5	Bal.	4.8	3.2	2.0	31.0
Plate-like debris (II)	94.8	5.2		0.7	0.6	-	6.1
Irregular blocky/Powder-like debris (III)	76.9	23.1		4.2	2.7	0.9	29.1
Intermetallic	N/A	N/A		42.8	29.1	7.4	-

## Data Availability

Not applicable.

## Supplementary Materials

The following supporting information can be downloaded at: https://www.mdpi.com/article/10.3390/ma16031193/s1, Figure S1: Hardness of Cu86.5 alloy after aging treatment. Figure S2: SEM images of Cu86.5 alloy after aging treatment.

## References

[1] Davis J.R. (2001). Copper and Copper Alloys.

[2] Xie G., Wang Q., Mi X., Xiong B., Peng L. (2012). The precipitation behavior and strengthening of a Cu–2.0wt% Be alloy. Mater. Sci. Eng. A.

[3] Kamikawa N., Sato K., Miyamoto G., Murayama M., Sekido N., Tsuzaki K., Furuhara T. (2015). Stress–strain behavior of ferrite and bainite with nano-precipitation in low carbon steels. Acta Mater..

[4] Zhang X., Zhang Y., Tian B., Song K., Liu P., Jia Y., Chen X., An J., Zhao Z., Liu Y. (2019). Review of nano-phase effects in high strength and conductivity copper alloys. Nanotechnol. Rev..

[5] Courtney T.H. (2005). Mechanical Behavior of Materials.

[6] Nembach E. (1997). Particle strengthening of metals and alloys. Mater. Sci. Technol..

[7] Basinski S., Basinski Z. (1980). Plastic deformation and work hardening. Dislocat. Solids.

[8] Gleiter H., Hornbogen E. (1968). Precipitation hardening by coherent particles. Mater. Sci. Eng..

[9] Wen H., Topping T.D., Isheim D., Seidman D.N., Lavernia E.J. (2013). Strengthening mechanisms in a high-strength bulk nanostructured Cu–Zn–Al alloy processed via cryomilling and spark plasma sintering. Acta Mater..

[10] Fan Z. (1995). A new approach to the electrical resistivity of two-phase composites. Acta Metall. Mater..

[11] Botcharova E., Freudenberger J., Schultz L. (2006). Mechanical and electrical properties of mechanically alloyed nanocrystalline Cu–Nb alloys. Acta Mater..

[12] Qu L., Wang E., Han K., Zuo X., Zhang L., Jia P., He J. (2013). Studies of electrical resistivity of an annealed Cu-Fe composite. J. Appl. Phys..

[13] Tian L., Anderson I., Riedemann T., Russell A. (2014). Modeling the electrical resistivity of deformation processed metal–metal composites. Acta Mater..

[14] Raeisinia B., Poole W.J., Lloyd D.J. (2006). Examination of precipitation in the aluminum alloy AA6111 using electrical resistivity measurements. Mater. Sci. Eng. A.

[15] Chuang M.-H., Tsai M.-H., Tsai C.-W., Yang N.-H., Chang S.-Y., Yeh J.-W., Chen S.-K., Lin S.-J. (2013). Intrinsic surface hardening and precipitation kinetics of Al0.3CrFe1.5MnNi0.5 multi-component alloy. J. Alloys Compd..

[16] Smith W.F. (1993). Structure and Properties of Engineering Alloys.

[17] Masamichi M., Yoshikiyo O. (1994). Effects of Co, Ni and Ti additions on the cellular precipitation in Cu-2% Be alloy. Mater. Trans. JIM.

[18] Buggy M., Conlon C. (2004). Material selection in the design of electrical connectors. J. Mater. Process. Technol..

[19] Schuler C.R., Kent M.S., Deubner D.C., Berakis M.T., McCawley M., Henneberger P.K., Rossman M.D., Kreiss K. (2005). Process-related risk of beryllium sensitization and disease in a copper–beryllium alloy facility. Am. J. Ind. Med..

[20] Kuhn H., Altenberger I., Käufler A., Hölzl H., Fünfer M. (2012). Properties of high performance alloys for electromechanical connectors. Copper Alloys: Early Applications and Current Performance—Enhancing Processes.

[21] Dong Q.-y., Shen L.-n., Wang M.-p., Jia Y.-l., Zhou L., Feng C., Chang C. (2015). Microstructure and properties of Cu–2.3 Fe–0.03 P alloy during thermomechanical treatments. Trans. Nonferrous Met. Soc. China.

[22] Wang W., Zou C.L., Li R.G., Wen W., Kang H.J., Wang T.M. (2016). In Situ Synchrotron X-ray Diffraction Study of a Deformed Cu-Fe-P Alloy during Heating. Materials Science Forum.

[23] Xiao X., Xu H., Chen J., Liang Q., Wang J., Zhang J. (2017). Aging properties and precipitates analysis of Cu–2.3 Fe–0.03 P alloy by thermomechanical treatments. Mater. Res. Express.

[24] Lei Q., Li Z., Dai C., Wang J., Chen X., Xie J.M., Yang W.W., Chen D.L. (2013). Effect of aluminum on microstructure and property of Cu–Ni–Si alloys. Mater. Sci. Eng. A.

[25] Lei Q., Li Z., Gao Y., Peng X., Derby B. (2017). Microstructure and mechanical properties of a high strength Cu-Ni-Si alloy treated by combined aging processes. J. Alloys Compd..

[26] Ye Y., Yang X., Liu C., Shen Y., Zhang X., Sakai T. (2014). Enhancement of strength and ductility of Cu–Sn–Zn alloy by iron addition. Mater. Sci. Eng. A.

[27] Gao W., Cao D., Jin Y., Zhou X., Cheng G., Wang Y. (2018). Microstructure and properties of Cu-Sn-Zn-TiO_2_ nano-composite coatings on mild steel. Surf. Coat. Technol..

[28] Fuxiang H., Jusheng M., Honglong N., Zhiting G., Chao L., Shumei G., Xuetao Y., Tao W., Hong L., Huafen L. (2003). Analysis of phases in a Cu–Cr–Zr alloy. Scr. Mater..

[29] Liu Q., Zhang X., Ge Y., Wang J., Cui J.-Z. (2006). Effect of processing and heat treatment on behavior of Cu-Cr-Zr alloys to railway contact wire. Metall. Mater. Trans. A.

[30] Su J., Liu P., Li H., Ren F., Dong Q. (2007). Phase transformation in Cu–Cr–Zr–Mg alloy. Mater. Lett..

[31] Zhao D., Dong Q.M., Liu P., Kang B.X., Huang J.L., Jin Z.H. (2003). Aging behavior of Cu–Ni–Si alloy. Mater. Sci. Eng. A.

[32] Yang B., Wu M., Li X., Zhang J., Wang H. (2018). Effects of cold working and corrosion on fatigue properties and fracture behaviors of precipitate strengthened Cu-Ni-Si alloy. Int. J. Fatigue.

[33] Lockyer S.A., Noble F.W. (1994). Precipitate structure in a Cu-Ni-Si alloy. J. Mater. Sci..

[34] Hu T., Chen J.H., Liu J.Z., Liu Z.R., Wu C.L. (2013). The crystallographic and morphological evolution of the strengthening precipitates in Cu–Ni–Si alloys. Acta Mater..

[35] Wang W., Kang H., Chen Z., Chen Z., Zou C., Li R., Yin G., Wang T. (2016). Effects of Cr and Zr additions on microstructure and properties of Cu-Ni-Si alloys. Mater. Sci. Eng. A.

[36] Zhang Y., Tian B., Volinsky A.A., Sun H., Chai Z., Liu P., Chen X., Liu Y. (2016). Microstructure and precipitate’s characterization of the Cu-Ni-Si-P alloy. J. Mater. Eng. Perform..

[37] Lei Q., Xiao Z., Hu W., Derby B., Li Z. (2017). Phase transformation behaviors and properties of a high strength Cu-Ni-Si alloy. Mater. Sci. Eng. A.

[38] Yi J., Jia Y., Zhao Y., Xiao Z., He K., Wang Q., Wang M., Li Z. (2019). Precipitation behavior of Cu-3.0 Ni-0.72 Si alloy. Acta Mater..

[39] Li J., Huang G., Mi X., Peng L., Xie H., Kang Y. (2019). Effect of Ni/Si Mass Ratio and Thermomechanical Treatment on the Microstructure and Properties of Cu-Ni-Si Alloys. Materials.

[40] Wang H.-S., Chen H.-G., Gu J.-W., Hsu C.-E., Wu C.-Y. (2015). Improvement in strength and thermal conductivity of powder metallurgy produced Cu–Ni–Si–Cr alloy by adjusting Ni/Si weight ratio and hot forging. J. Alloys Compd..

[41] Patchett J., Abbaschian G. (1985). Grain refinement of copper by the addition of iron and by electromagnetic stirring. Metall. Trans. B.

[42] Suzuki S., Shibutani N., Mimura K., Isshiki M., Waseda Y. (2006). Improvement in strength and electrical conductivity of Cu–Ni–Si alloys by aging and cold rolling. J. Alloys Compd..

[43] Lei Q., Li Z., Xiao T., Pang Y., Xiang Z.Q., Qiu W.T., Xiao Z. (2013). A new ultrahigh strength Cu–Ni–Si alloy. Intermetallics.

[44] Li Y., Xiao Z., Li Z., Zhou Z., Yang Z., Lei Q. (2017). Microstructure and properties of a novel Cu-Mg-Ca alloy with high strength and high electrical conductivity. J. Alloys Compd..

[45] Lee E., Han S., Euh K., Lim S., Kim S. (2011). Effect of Ti addition on tensile properties of Cu-Ni-Si alloys. Met. Mater. Int..

[46] Cheng J.Y., Tang B.B., Yu F.X., Shen B. (2014). Evaluation of nanoscaled precipitates in a Cu–Ni–Si–Cr alloy during aging. J. Alloys Compd..

[47] Zhang J., Lu Z., Zhao Y., Jia L., Xie H., Tao S. (2017). Disintegration of the net-shaped grain-boundary phase by multi-directional forging and its influence on the microstructure and properties of Cu–Ni–Si alloy. Mater. Res. Express.

[48] Cao Y., Han S.Z., Choi E.-A., Ahn J.H., Mi X., Lee S., Shin H., Kim S., Lee J. (2020). Effect of inclusion on strength and conductivity of Cu-Ni-Si alloys with discontinuous precipitation. J. Alloys Compd..

[49] Xie H., Jia L., Lu Z. (2009). Microstructure and solidification behavior of Cu–Ni–Si alloys. Mater. Charact..

[50] Jia Y.-l., Wang M.-p., Chen C., Dong Q.-y., Wang S., Li Z. (2013). Orientation and diffraction patterns of δ-Ni_2_Si precipitates in Cu–Ni–Si alloy. J. Alloys Compd..

[51] Archard J.F. (1953). Contact and Rubbing of Flat Surfaces. J. Appl. Phys..

[52] Purcek G., Yanar H., Saray O., Karaman I., Maier H.J. (2014). Effect of precipitation on mechanical and wear properties of ultrafine-grained Cu–Cr–Zr alloy. Wear.

[53] Stott F. (1998). The role of oxidation in the wear of alloys. Tribol. Int..

[54] Khodabakhshi A., Abouei V., Mortazavi N., Razavi S.H., Hooshyar H., Esmaily M. (2015). Effects of cold working and heat treatment on microstructure and wear behaviour of Cu–Be alloy C17200. Tribol.-Mater. Surf. Interfaces.

