# Supplementary material for: Investigation of Microstructure and Wear Properties of Precipitates-Strengthened Cu-Ni-Si-Fe Alloy
Source: Materials (Basel). 2023 Jan 30;16(3):1193. doi: 10.3390/ma16031193 (PMC9921433; doi:10.3390/ma16031193)
Supplement: Supplementary file 1 [file materials-16-01193-s001.zip › materials-2152579-supplementary.pdf]

Supplemental Material

The hardness and microstructure of Cu86.5 alloy with different aging treatment.

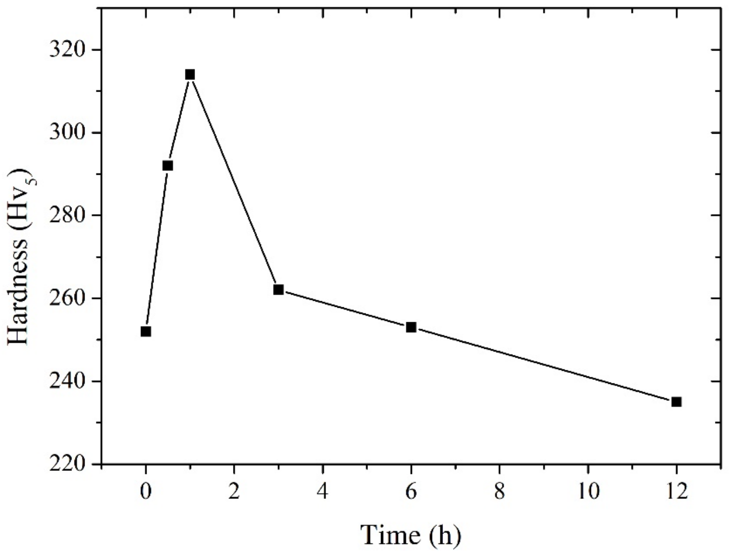

Figure S1. Hardness of Cu86.5 alloy after aging treatment

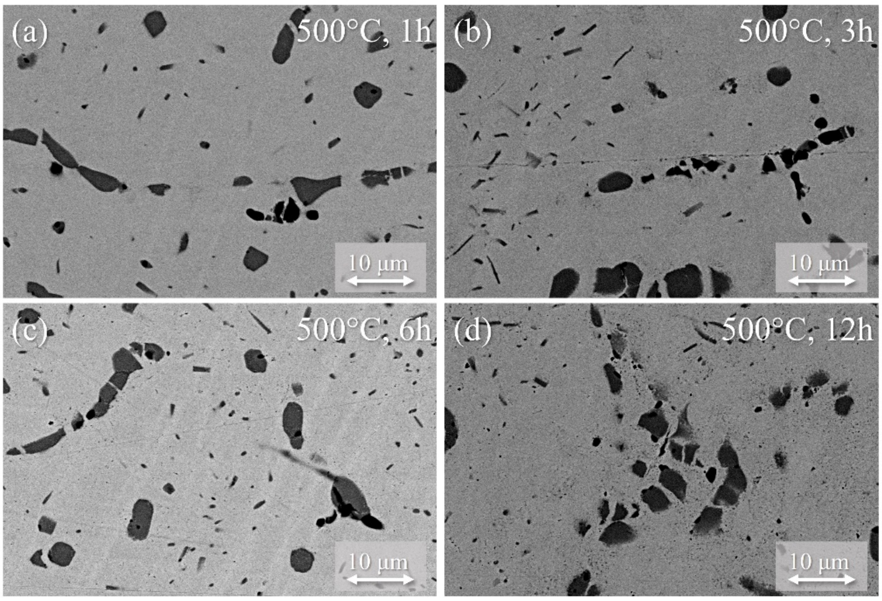

Figure S2. SEM images of Cu86.5 alloy after aging treatment
